# What supports mothers of very preterm babies to start and continue breast milk feeding neonatal units? A qualitative COM-B analysis of mothers’ experiences

**DOI:** 10.1186/s12884-024-06910-4

**Published:** 2024-11-06

**Authors:** Jenny McLeish, Annie Aloysius, Chris Gale, Maria A. Quigley, Jennifer J. Kurinczuk, Fiona Alderdice

**Affiliations:** 1 Nuffield Department of Public Health, NIHR Policy Research Unit in Maternal and Neonatal Health and Care, National Perinatal Epidemiology Unit, University of Oxford, Old Road Campus, Headington, Oxford, OX3 7LF UK; 2 Imperial College Healthcare NHS Trust, London, UK; 3 School of Public Health, Faculty of Medicine, Neonatal Medicine, Imperial College London, Chelsea and Westminster Hospital Campus, London, SW10 9NH UK

**Keywords:** Neonatal unit, Very preterm, Breastfeeding, Expressing, Qualitative, Mothers’ experiences, COM-B

## Abstract

**Background:**

It is challenging for mothers who give birth very preterm to produce sufficient breast milk by expressing for weeks before their baby is able to feed from the breast, and then to transition from tube feeding to breastfeeding. Lactation is most successful when stimulated shortly after birth, established within 72 h, and maintained by expressing 6–8 times a day. This study explored mothers’ experiences of how breast milk feeding and breastfeeding for very preterm babies can be supported by staff and the facilities of a neonatal unit.

**Methods:**

Twenty-three mothers of very preterm babies were interviewed, from four neonatal units in England with high or low rates of breast milk feeding at discharge. Interviews were analysed using the COM-B framework to consider how mothers’ behaviour (breast milk feeding and breastfeeding) is affected by capability, opportunity and motivation.

**Results:**

Mothers’ motivation in the traumatic situation of very preterm birth was strongly affected by information from staff about the benefits of breast milk for their baby, the importance of early and frequent expressing, and how to assess the effectiveness of direct breastfeeding. It was maintained through positive feedback about their efforts which built their confidence, and reassurance about what is ‘normal’ growth when a baby begins direct breastfeeding. Motivation needed to be supported by opportunity, including access to equipment for expressing and facilities to stay near the neonatal unit, and also by capability, which required proactive and skilled information and support from staff. Specialist support and facilities varied between units, and some mothers were not given necessary information or had their motivation undermined by staff comments.

**Conclusions:**

Interventions to increase breast milk feeding and breastfeeding for very preterm babies should address mothers’ motivation, capability and opportunity, aiming for systematic elimination of obstacles. Mothers value personalised and skilled specialist support, but also need other staff to be able to give consistent information and affirmation focused on their efforts rather than their success, with a trauma-informed approach. Investing in rooming-in facilities that minimise the separation of mothers and babies is likely to overcome a key obstacle.

**Supplementary Information:**

The online version contains supplementary material available at 10.1186/s12884-024-06910-4.

## Background

Maternal breast milk is a key intervention to reduce the risk of adverse physical and neurodevelopmental outcomes for babies born preterm [1–3]. ‘Very preterm’ babies (defined by the World Health Organisation as those born before 32 weeks gestation [4]) are the most vulnerable but are also less likely to receive their mother’s breast milk [5, 6]. Very preterm babies lack the muscle tone and endurance to feed directly from the mother’s breast and are unable to co-ordinate sucking, swallowing and breathing. They are fed milk by orogastric or nasogastric tube until they are developmentally ready for direct oral feeding from the breast or by bottle or cup, which may be in combination with ongoing tube feeding [7, 8].

Feeding a very preterm baby with the mother’s breast milk from birth can be challenging because production of breast milk may be delayed, and she may have to maintain lactation by expressing milk for many weeks before the baby is able to feed from the breast [8, 9]. Lactation is most successful if it is stimulated very shortly after birth, established within 72 h, and maintained by expressing 6–8 times a day; this early initiation and frequent pumping being associated with higher volumes of milk production and longer provision of breast milk by mothers of preterm babies [9–15]. Establishing exclusive direct breastfeeding is important for preterm babies as this is associated with longer term breast milk feeding after leaving the neonatal unit [10, 16], but is challenging for mothers who are separated from their babies [10, 17, 18]. The average length of stay in neonatal units in England is 123 days for babies born at 24 weeks gestation and 34 days for babies born at 31 weeks gestation [19].

There is a substantial literature exploring mothers’ feelings about feeding their preterm babies and its complex interaction with the development of maternal identity for mothers whose babies are on a neonatal unit. Mothers who successfully expressed their milk describe this as important for their confidence, attachment, overcoming feelings of guilt that their bodies had failed, and the development of their maternal role; but expressing milk is also experienced as an exhausting and stressful obligation and if it is unsuccessful, this can trigger increased feelings of guilt and shame [20–23]. Mothers describe motivation, information and support from staff as very important, as well as the provision of facilities by the neonatal unit for mothers to express their milk or stay with their babies [20–23]. In one review mothers’ experiences were analysed through the theoretical framework of coping [21] and one study used the lens of self-efficacy [24], but there has otherwise been limited use of theory to understand feeding experiences on neonatal units and none for very preterm babies.

**Fig. 1:**
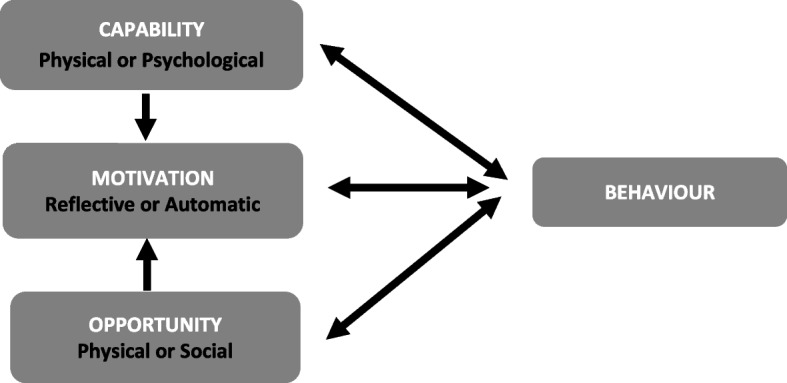
The COM-B model - a framework for understanding behaviour [25]

Interventions to change behaviour are often implemented without an analysis of the complex factors that may affect their success [25]. Understanding the factors affecting behaviour offers a basis for designing effective interventions to achieve behaviour change. The COM-B model [25] is a framework for understanding behaviour change, based on the insight that any behaviour (B) requires three interacting factors: capability (C), opportunity (O), and motivation (M) (see Fig. 1). The aim of this study was to generate insight using the COM-B model, based on mothers’ experiences, into how breast milk feeding and breastfeeding for very preterm babies can be supported by staff and the facilities of a neonatal unit, with the intention of enabling future interventions for this population to be developed and delivered more effectively. This study is part of a programme of work that also explored health professionals’ experiences of giving support (reported separately).

**Table:** 

Definitions for Fig. 1 as applicable to breast milk feeding for very preterm babies
Capability (C): the ability to carry out the behaviour. This could be physical capability (e.g. the physical ability of the mother to produce and express breast milk) or psychological capability (e.g. knowledge of how to express or breastfeed).
Opportunity (O): external factors that make the behaviour possible. This could be physical opportunity (e.g. having the time, equipment or access to facilities to express or breastfeed) or social opportunity (e.g. expressing and breastfeeding is a cultural norm).
Motivation (M): the internal brain processes that affect behaviour. This could be reflective motivation (e.g. a conscious desire to express or breastfeed) or automatic motivation (e.g. fear for the baby).
For an intervention to affect a person’s behaviour, it must first change one or more of the other factors, which can also influence each other. Behaviour can also affect the other factors, such as a positive or negative feedback loop between successfully or unsuccessfully performing a behaviour and feeling motivated to continue it [25].

## Methods

This was a qualitative interview-based study using the COM-B model, theoretically informed by phenomenological social psychology [26], acknowledging the role of both participants’ understandings and the researchers’ interpretations in the production of knowledge [27]. Four neonatal units in England were purposively selected based on their rates of breast milk feeding for very preterm babies at discharge [28], two with higher than average rates of over 75% (called Units A and B in this article) and two with lower than average rates of below 40% (called Units C and D in this article). Units B and D provided tertiary level care for babies of all gestations and prolonged intensive care (called Neonatal Intensive Care Units or tertiary units). Units A and C provided initial care for babies down to 27–28 gestational weeks and short periods of intensive care (called Local Neonatal Units or non-tertiary units) [29]. In the wake of previous Covid-19 restrictions [30], during the time that the babies in this study were receiving neonatal care (between December 2020 and December 2022) all four units allowed parents unrestricted time with their babies but no other children or family members could visit.

To recruit a sample based on maximum diversity of feeding experience, mother’s age and ethnicity, a key contact at each neonatal unit invited mothers of very preterm babies to participate in the study and passed on the contact details of those who agreed. The inclusion criteria were that the mother had given birth to a baby or babies at less than 33 weeks gestation, she had expressed milk and/or breastfed her baby for any length of time on the neonatal unit, and the babies were either still on the neonatal unit or had been discharged in the last 12 months. The gestation of < 33 weeks reflected the cut-off used by England’s National Neonatal Audit Programme to report on breast milk feeding for very preterm babies at the time the study began [31], which differs from the more commonly used World Health Organisation definition of < 32 weeks [4]. Professional interpreting was offered if required, but all participants chose to be interviewed in English.

Participant information and consent forms were emailed to participants at least 24 h in advance; informed consent was obtained at the beginning of the interview. Each participant took part in a single semi-structured telephone interview between March and December 2022. Interview topic guides were developed with the support of a Parent, Patient and Public Involvement (PPPI) group (see Additional File 1). Recruitment continued until data saturation was reached (23 participants)—that is, participants were repeating what had been expressed by previous participants and there were no new codes or themes. A further six mothers had declined to participate when contacted or were uncontactable.

Interviews were audio-recorded and transcribed by a professional transcription service. Transcripts were checked against audio-recordings and reread for familiarity. Transcripts were analysed using the COM-B framework to guide coding, which was both deductive (based on the literature) and inductive (responding to new points raised by interviewees). Codes were separately recorded using NVIVO software for each of the five key stages of the breast milk feeding journey in the neonatal unit—deciding to feed breast milk, starting expressing, maintaining expressing, the transition to direct breastfeeding, and going home. Codes were combined and developed into a structure reflecting COM-B sources of behaviour. JM analysed all transcripts and FA and AA each analysed a subset; codes and the resulting COM-B structure were discussed and agreed. The researchers had no prior relationship with interviewees, and reflected critically on their own varied personal experiences of infant feeding and professional experiences of working in neonatal units caring for very preterm babies and their parents.

## Results

### Participants

Twenty three mothers took part in interviews. There were 25 babies—21 singletons and two sets of twins—born at gestations between 24 weeks 1 day and 32 weeks 4 days. Eleven mothers had given birth to extremely preterm babies (before 28 weeks gestation). Three mothers had prior experience of a very preterm birth or stillbirth in addition to their current experience. Mothers’ demographic characteristics and aspects of their birth and feeding experiences are shown in Table 1. The characteristics of the neonatal units where the babies had most recently received care are shown in Table 2.

**Table 1:** Mother’s demographic characteristics, birth and feeding experiences

Characteristics and experiences	Number of mothers (*n* = 23)
**Age**	
< 20	1
20–24	2
25–29	4
30–34	6
35–40	7
41 +	3
**Parity**	
1st child/ren	14
2nd or subsequent child/ren	9
**Ethnicity**	
Asian	4
Black	4
Chinese	1
White	14
**Partnership**	
With partner	22
Single parent	1
**Babies’ gestation at birth**	
24 + 0 - 25 + 6	4
26 + 0 - 27 + 6	6
28 + 0 - 29 + 6	4
30 + 0 - 31 + 6	5
32 + 0 - 32 + 6	4
**Place of birth**	
Same hospital as current neonatal unit	13
Different hospital	9
At home	1
**Feeding intention before very preterm birth**	
Breastfeed (definite)	11
Breastfeed (tentative)	10
Formula	1
Had not thought about it	1
**Situation at time of interview**	
On neonatal unit	10
At home ≤ 1 month	8
At home > 1 month	5

**Table 2:** Participating units and interviewees

Unit pseudonym	Level of neonatal care	Specialist infant feeding support	Rate of very preterm babies receiving breast milk at discharge, compared to England average*	Mothers’ pseudonyms
Unit A	Local Neonatal Unit	Infant feeding nurse – 2 days per week	Higher rate	M04, M05, M07, M13, M17
Unit B	Neonatal Intensive Care Unit	Infant feeding nurse – full time	Higher rate	M02, M03, M08, M09, M14, M15
Unit C	Local Neonatal Unit	Infant feeding midwife from postnatal ward if available	Lower rate	M01, M06, M10, M11, M12, M16
Unit D	Neonatal Intensive Care Unit	Infant feeding nurse – 1 day per week	Lower rate	M18, M19, M20, M21, M22, M23

^*^Based on figures from the National Neonatal Audit Programme [28]

### Findings

**Fig. 2:**
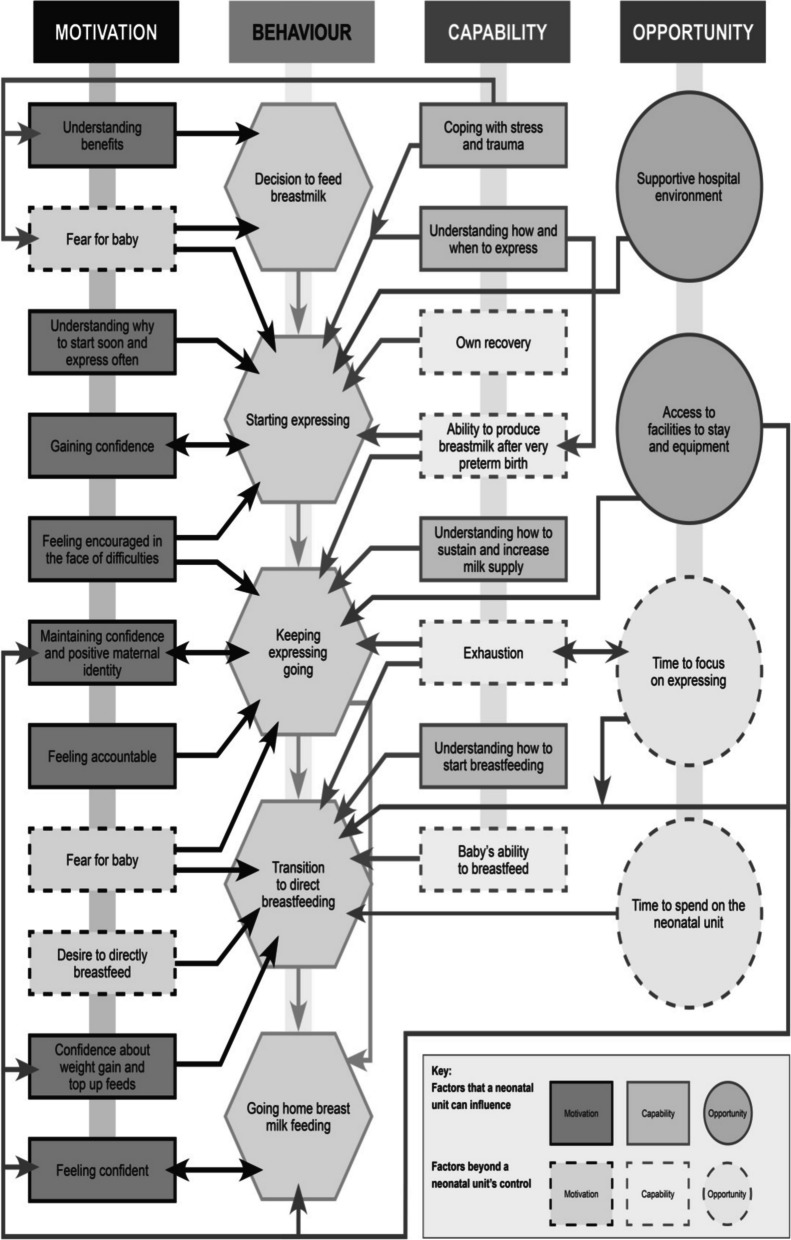
COM-B analysis of factors affecting mothers’ breast milk feeding of very preterm babies

The findings are presented as capability (C), opportunity (O) and motivation (M) factors affecting five key behaviours (B) during a very preterm baby’s time on a neonatal unit: the decision to feed breast milk, starting expressing, keeping expressing going, the transition to direct breastfeeding, and going home breastfeeding or breast milk feeding. Fig. 2 shows the interaction of factors affecting mothers’ capability, opportunity, motivation and behaviour. Table 3 shows the interventions on the neonatal unit which mothers identified as supporting their capability, opportunity, and motivation.

**Table 3:** Issues, interventions and obstacles affecting capability, opportunity and motivation

Behaviour	Issue	Capability (C) opportunity (O) or motivation (M)	Interventions	Obstacles
Deciding to feed breast milk	No intention to breastfeed or tentative intention to breastfeed	Understanding benefits (M)	Antenatal or postnatal information about benefits of breast milk for very preterm babies	Lack of trauma-informed approach
Deciding to feed breast milk	Difficult to take in information before and after very preterm birth Baby in fragile condition	Coping with stress and trauma (C) Fear of losing baby (M)	Acknowledgement of trauma, signposting to support	
Getting expressing started	Unaware of importance of starting at once Unaware milk produced so early	Understanding why to start soon and express often (M) Coping with stress and trauma (C)	Clear, timely information Acknowledgement of trauma, signposting to support	Difficult to take in information before and after very preterm birth Lack of trauma-informed approach Inconsistent advice if no specialist available
Getting expressing started	Unaware how to express Some mothers prefer to hand express colostrum, others prefer to use a pump	Understanding how to express (C)	Clear, consistent information and practical help to get started Information and help given proactively Reassurance that colostrum is produced in tiny amounts Offering choice of expressing by hand or with a pump	No system to ensure mothers have information Contradictory information from staff on neonatal unit, or between neonatal unit and postnatal ward Postnatal staff unaware how to support mothers after very preterm birth Mothers do not feel able to ask for help and staff are too busy to offer it No designated member of staff to support feeding
Getting expressing started	Need for a comfortable place to express milk Lack of physical contact with baby inhibits production of colostrum and breast milk	Hospital environment (O)	Information about benefits of expressing beside the baby Comfortable space beside the cot Assistance to get to neonatal unit from postnatal ward Information about other ways to bring baby to mind when separated	Postnatal ward not conducive to expressing Postnatal staff too busy to assist mother to get to neonatal unit
Getting expressing started	Lack of confidence	Gaining confidence (M)	Affirmation Being made to feel like a valued part of the neonatal team	
Getting expressing started	Demoralised when there are difficulties	Feeling encouraged (M)	Encouragement to persist, reassurance that stimulation is more important than volume	Mother afraid to get attached to baby Critical or unsupportive staff comments
Getting expressing started	Baby in fragile condition	Fear of losing baby (M)	Acknowledgement of trauma, signposting to support	Lack of trauma-informed approach
Keeping expressing going	Regular pumping needed to maintain milk supply Mother has other children to look after	Time to focus on expressing (O)	Support to hold baby skin-to-skin while pumping Affirmation Acknowledgement of challenges	
Keeping expressing going	Lack of physical contact with baby inhibits production of breast milk Mother lives far from unit	Facilities to stay on or near unit (O)	Parent accommodation provided near neonatal unit	
Keeping expressing going	Unaware of ways to sustain and increase milk supply	Understanding how to sustain and increase milk supply (C)	Information about ways to sustain and increase milk supply Information given proactively	Anecdotal suggestions from nurses Mothers do not feel able to ask for help and staff are too busy to offer it
Keeping expressing going	Need for a comfortable place to express milk	Neonatal unit facilities (O)	Comfortable space beside the cot Comfortable expressing room	
Keeping expressing going	Need for high quality electric breast pump	Neonatal unit equipment (O)	Electric breast pumps available on ward Free loan of electric breast pumps	Loaned pumps not returned by parents and no system for follow up
Keeping expressing going	Regular pumping needed to maintain milk supply Lack of physical contact with baby inhibits production of breast milk	Time to focus on expressing (O)	Mothers allowed to spend as much time on unit as they want Support to hold baby skin-to-skin while pumping Parent accommodation provided near neonatal unit Acknowledgement of challenges and affirmation	
Keeping expressing going	Maintaining confidence	Remaining confident and positive maternal identity (M)	Being made to feel like a valued part of the neonatal team	
Keeping expressing going	Demoralised when there are practical difficulties Mothers are exhausted by round-the- clock expressing and visiting the neonatal unit	Feeling encouraged in the face of difficulties(M)	Encouragement to persist, praise for efforts made ‘Permission’ to prioritise self-care	Seeing other mothers’ higher milk volume intensifies feelings of failure
Keeping expressing going	Baby in fragile condition	Fear of losing baby (M)	Acknowledgement of trauma, signposting to support	Lack of trauma-informed approach
Keeping expressing going	Temptation to do less than recommended	Feeling accountable (M)	Accountability and non-judgemental honest feedback	Mothers doubt sincerity of constant praise
Transition to direct breastfeeding	Unaware when and how to introduce direct breastfeeding	Understanding when and how to start breastfeeding (C)	Proactive information and support	Inconsistent advice if no specialist available
Transition to direct breastfeeding	Strain of combining expressing and breastfeeding	Coping with exhaustion (C)	Encouragement to persist, praise for efforts made	
Transition to direct breastfeeding	Baby in fragile condition	Fear of losing baby (M)	Acknowledgement of trauma, signposting to support	Lack of trauma-informed approach
Transition to direct breastfeeding	Separation from the baby inhibits opportunities to breastfeed	Spending time on the neonatal unit (O)	Parent accommodation near or on neonatal unit	Staff want to respond to feeding cues by bottlefeeding Inflexible tube feeding schedule Discharge criteria require cessation of tube feeding but mother not offered accommodation on unit to establish full breastfeeding
Transition to direct breastfeeding	Anxiety about volume of milk taken, volume of top-up tube feeds, and baby’s weight	Confidence about weight gain and managing top-up feeds (M)	Reassurance about normal weight loss Peer support Flexibility about top ups	Critical or unsupportive comments Inflexible discharge protocol
Going home breast milk feeding	Lack of confidence	Feeling confident to go home breast milk feeding (M)	Information Parent accommodation within neonatal unit Ongoing support from outreach team	
After discharge	Strain of combining expressing and childcare Weight loss when human milk fortifier stopped Unable to increase milk supply as baby’s needs increase	Sustaining breast milk feeding after discharge (C)	Transition to breastfeeding before discharge Ongoing support from outreach team ‘Permission’ to stop expressing	

## Deciding to feed breast milk

### Understanding the benefits of breast milk for very preterm babies

Most of the mothers had already formed an intention to try breastfeeding before they realised their baby was going to be born very preterm. Their motivation was strengthened by receiving information about the specific benefits of breast milk for very preterm babies and understanding that this might be the one thing they could do to help their newborn’s chance of survival (M). One mother had not breastfed her other children but was persuaded to try expressing her milk by information from staff:

*“They said ‘You don’t have to if you don’t want to, but because she’s premature it’s like gold dust to her’. So I said ‘Well if it’s gonna help her then I will, I’ll do anything to make her still be here now.’”* M06.

### Coping with stress, trauma and fear

Mothers’ motivation might be undercut by the impact of trauma, fear and stress on their psychological capability to process information and make decisions about feeding (C). There was no consensus about the optimal time to receive the information about feeding. Although a couple of mothers appreciated the chance to learn about expressing milk during an antenatal inpatient stay, others said that an antenatal conversation about feeding would have been meaningless when they were overwhelmed with worry about unexpected preterm birth:

*“It was, ‘Will he survive?’ So when I went in [to hospital] at 23 weeks it was, ‘We can try and save him, or you can make memories.’ … So it was very complicated, and the thought of how do you feed him didn’t even come into my mind.”* M20.

Likewise some mothers said that after giving birth to a very preterm baby they were so stressed that it was difficult to take in information about feeding options postnatally, and fear that the baby would not survive might inhibit a decision to feed breast milk (M):

*“I was so exhausted and so out of it [after birth] … [I thought]* ‘*Do I need to go get emotionally attached to what isn’t going to be?’ … I’m sure people were saying things, but I don’t know what they were saying.”* M08.

Although staff could not take away the intense stress mothers experienced, their response could affect how well mothers coped and thus their psychological capability. One mother described how she was given *“perfunctory”* instructions about expressing milk shortly after an unexpected extreme preterm birth. She felt she needed someone to address the traumatic experience she had just been through, before she could begin to engage with the reality of being a mother.

*“I’m just thinking, ‘Okay then, we made it, we’re alive’… At that point somebody said, ‘Are you planning on breastfeeding?’ and I suddenly felt like I’d been shifted into a different world. And people kept on calling me, ‘Do you want to do this, mum? Do you want to do that, mum?’ and I kept looking round thinking, ‘My mum’s not here, why are they calling my mum?’ … And they didn’t acknowledge the trauma. I nearly died … I needed some kind of acknowledgement, not just slip into the normal flow of things. It didn’t feel natural, it didn’t feel normal, it felt very, very artificial and very, very scary.”* M20.

## Getting expressing started

### Understanding why and how to start expressing soon and often

Mothers were not necessarily aware that their bodies were capable of producing breast milk after a very preterm birth. Timely information from staff was needed for them to understand the importance of starting to express colostrum in the first hours after giving birth, to stimulate the production of breast milk (M). They were generally encouraged to express at least 8 times in every 24 h, including at least once during the night between 2 a.m. and 4 a.m..

*“After the [caesarean] section they explained the importance, with the baby being so small, to get the colostrum if we can…I didn’t think my body would be able to do it with it being so early, and [the nurse] said, ‘Yes, we can do it, you’ve just got to be consistent and try and not worry if you don’t get anything.’”* M16.

Mothers also needed information about *how* to express colostrum (C), either using syringes to catch the drops of colostrum expressed by hand, or using a breast pump. Most mothers were encouraged to express their colostrum by hand and then transfer to an electric breast pump after a few days when their breast milk came in, while some were advised to use a breast pump immediately. Expressing by hand and using a pump were unfamiliar skills for most mothers and they found it was helpful to be *shown* as well as *told* how to express. Even if they understood the techniques, some also needed practical help to manage the syringes for hand expressing (C):

*“I was so tired and emotional, exhausted, I was getting myself a bit stressed … So I did the massage and the squeeze and then [the midwife] did the catching it with the syringe which just made things so much easier.”* M15.

A few mothers (at all four units) had the opposite experience, where they were not told about the importance of early and frequent expressing to stimulate their milk supply (M), or were not given the skilled help they needed to get started (C) because of staff shortages on the postnatal ward or because their needs as mothers of very preterm babies were not recognised by postnatal staff:

“*I didn’t know I had to express every three hours or even more, so it would be only once or two times a day that I would express.”* M13.

*“The day after I had [my baby] this midwife came in with this pump and was like, ‘You need to get attached to this, you need to get milk for your baby,’ I just felt it was thrown at me… I gave it a go but nothing was happening … and she was like, ‘Well, you must have been doing it wrong then.’ When other midwives come in they’d be like, ‘Is your baby on NICU?,’ and you’d explain your story for the 100th time that day…Maybe if that had been passed around better their approach would have been different.”* M03.

Mothers highlighted their absolute dependence on staff in the wholly new situation of having a very preterm baby in neonatal care, unaware of what information or support was available unless staff told them. Several described their distress at being given contradictory information about how to express, which affected their confidence (M) as well as their physical capability to establish a milk supply (C).

*“I was hand expressing for about five days after delivery. [Then] the visiting doctor for the baby told me, ‘You should be expressing properly [with a pump] now, you should have been doing that for days’… Someone told me that if after the 15 min I didn’t have a flow of milk I should continue [pumping], and then I was doing that for a couple of days and my breasts got very engorged, so another nurse said I shouldn’t be doing that because I’m giving my body the wrong signal how much milk I need…After we’d been there for a couple of weeks they told us that we should have gotten all this information, all those leaflets and bits and bobs at the beginning … and obviously I didn’t know what I needed to have; I didn’t know what to expect, to ask for it either.”* M21.

Mothers at Units C and D noted that the potential for inconsistent advice was increased where they were not aware of any designated member of staff on the neonatal unit as the first point of contact for feeding questions:

*“There’s not a specialist there for [feeding]. You could ask one nurse about one thing, and they can give you an answer, and ask another nurse the same question and they give you an inconsistent answer… So I google everything. The Holy Grail, Google.”* M10.

Mothers from Unit C had additionally been confused by disagreement about the best method of starting to express, between the midwives who were looking after them on the postnatal ward and the staff looking after their babies on the neonatal unit:

*“NICU tell you, ‘Pump from day 1, you need to get the colostrum … and [staff on the postnatal ward] were like, ‘You don’t really need to do it… You just need to hand express’ … I went with what the NICU were saying because at the end of the day they specialise in it.”* M11.

### Physical recovery and the hospital environment

The early days were complicated by mothers’ exhaustion and need to recover from birth, affecting their physical capability (C). This meant that despite high motivation, they could not necessarily spend much time with their baby while staying on the postnatal ward, or express on the demanding timetable that was recommended.

*“I was doing what I could do but it was nowhere near eight times a day, or even six, or five … in between being in pain down below and being tired and just trying to work out what is going on.”* M08.

The physical environment of the postnatal ward and neonatal unit (O) also affected mother’s ability to establish expressing. The postnatal ward full of other mothers’ babies was not conducive to the relaxation necessary for milk flow (O). Mothers were encouraged to express beside their baby’s cot on the neonatal unit instead, and being an in-patient at the same hospital potentially enabled them to do this at any time if they felt able (O):

*“The last place you want to be is on a [postnatal] ward with loads of babies when your baby’s stuck downstairs in an incubator… And then that makes it hard to pump because you don’t feel relaxed at all. [The neonatal nurses] suggested to me to pump by the side of his incubator, because they said it helps, and it did, it was best thing I could do.”* M11.

However, mothers who were themselves unwell or recovering from caesarean birth could not always do this, because they were in too much pain (C), or the assistance they needed to move between the postnatal ward and the neonatal unit was lacking (O).

*“I was really ill … I only had a quite uncomfortable chair by his incubator, so I physically couldn’t stay there for very long. To begin with I could only manage about an hour and then I was in a lot of pain and had to go and lie down, so I expressed when I was in the bed [on the postnatal ward].”* M22.

Several mothers had been told that when they could not be with their baby, they could stimulate the flow of milk by looking at pictures of their baby or smelling a ‘bonding square’ that had been placed in the incubator. Some said that these techniques had appeared to work for them, others that they did not.

### Gaining confidence through affirmation and success

Where a mother succeeded in expressing colostrum, affirmation from healthcare professionals could help her confidence which motivated her to continue her efforts (M).

*“There was a breastfeeding lady on the ward and she was brilliant, she came round to see me and she did some hand expressing with me… She was so positive about it and about how well I was doing, I had this kind of mental attitude that, ‘Oh! I* *can* *do this, the expert [says so].’”* M07.

Enthusiastic feedback could also encourage a mother to see herself as a valued member of the team, with a unique role in her baby’s care (M).

*“I felt like there was camaraderie there, when I was dropping off the colostrum at 12 midnight, and handing it over to the nurses and them saying, ‘Oh well done mummy, that’s great.’ Then I felt really good … I felt supported, I trusted them, and a lot of them just seemed so happy with it.”* M17.

Some mothers (including some who had experienced extreme preterm birth) described themselves as producing colostrum and then abundant milk without difficulty and this early ‘success’ set up a positive feedback loop of breastfeeding self-efficacy and motivation to follow the demanding schedule (M).

*“[Expressing colostrum] was a challenge but after two days … it fell into place and I was good with it. As soon as I started expressing literally it just started gushing out…They were telling me, ‘Make sure you eat food with loads of calories, make sure you express over the night, five times a day,’ I was like, ‘Right, if this is what’s going to make my son be good, let’s do it.’”* M12.

### Feeling encouraged in the face of practical and psychological difficulties

Some mothers found it demoralising when their efforts to start expressing initially produced almost nothing (C), and there was a risk of a negative feedback loop of ‘failure’ (M). The encouragement from staff that it was worthwhile persisting, because stimulating the breasts was more important than volumes of milk produced at the beginning, was critical for mothers who were full of self-reproach (M):

*“With her coming early I felt my body had totally let me down … If I then put all this pressure on, ‘Well why am I not producing milk?’ as well, it’s really difficult. But someone saying, ‘No, your body’s doing what it’s meant to be doing and you’re doing great, you just need to be kind to yourself,’ makes such a difference… It gave me the confidence to continue and think, ‘Okay, I’m not totally useless.’… I went on to produce so much milk, and I donated loads to the milk bank, and I’m sure it’s because I had that positive start at the beginning.”* M07.

Encouragement to persist was also crucial when a mother was struggling to deal with the trauma of what had happened, and wanted to protect herself psychologically from forming a bond with a baby whom she thought might die (M):

*“In the initial days I did not want myself to get attached to him … blocking my mind, ‘I don’t have no son, I don’t think he’s going to survive.’ So, I have had to fight all my thoughts… I had to force myself to sleep because there were so many things on my mind, and then if I have to wake myself up in the night [to express], every time I used to wake up the trauma used to hit me again…In the beginning it was just [nurses] who motivated me, ‘No you can do it, he needs your milk, it will come’.”* M01.

By contrast, some mothers at all units described having their motivation and self-belief undermined by casual unsupportive comments from staff.

*“That day I had expressed 30ml and that was the biggest I ever had that early on, and I remember feeling so happy and I took it in … [The nurse] said, ‘Oh, is that all you’ve got from one expression?’ I remember my heart sunk and I said, ‘Yes, that’s my first one, I’m really pleased with that,’ and she just looked at me and she was like, ‘You're not really trying though, are you?’ I remember just wanting to cry…. It must have been day 4 or day 5, and it really hurt me because I felt I had done so well.”* M03.

Several mothers reflected on the specific psychological vulnerability of mothers in the neonatal unit, and the importance of staff understanding that distressed and disorientated mothers may read unintended meanings into *“offhand”* comments, crushing their motivation and their ability to understand information accurately (C).

*“I was so hypersensitive as well because of everything that was going on, and it’s just little comments that people don’t think anything of, but unless you’ve been in the situation, you don’t quite realise the impact that using the wrong words could have.”* M07.

## Keeping expressing going

The mothers had expressed their milk for periods between 2 weeks and 14 months. Once they had been discharged from the postnatal ward, they expressed at home and brought the milk in to the neonatal unit, or expressed on the unit while visiting their baby.

### Time to focus on expressing and facilities to stay

When they were sufficiently well and their babies were stable, many of the mothers spent significant periods of time on the neonatal unit holding their babies skin-to-skin every day (O). They believed that this had helped them to produce milk, especially if they were supported to pump and hold their baby at the same time (although this was unusual):

*“When I used to hold them, 10 min later I would start leaking, I’d have a lot of milk then I’d have to go and express …I did give myself the time to do skin-to-skin with them, an hour at least with each, or two hours*.” M05.

Other mothers faced structural barriers to spending time expressing, or spending time with their babies which would help them to sustain their expressing. The most challenging issues were living a long distance from the neonatal unit, necessitating long and expensive journeys once the mother had been discharged from the postnatal ward; and having other children to look after and insufficient family support to share their care.

None of units was able to offer mothers the option to room-in with their babies 24 h a day (O). Some mothers had been able to stay in hospital-provided accommodation near the neonatal unit which made an enormous difference to their ability to spend time with their baby and to prioritise expressing (O).

*“I literally do the skin-to-skin about 8 h a day… It’s 50 min to get to the NICU [from home], so they moved me to [the on-site accommodation] … One of the nurses made the comment the other day to say that I visit [the on-site accommodation] and I stay at the NICU.”* M08.

Where this was not possible because there was no parent accommodation, or because their family commitments made it impractical, staff could not solve the problem but could support mothers by acknowledging the difficulties they faced and encouraging them to do whatever they could manage in the circumstances (M).

*“We had to learn to juggle things around my [older] son … we were spending about two and a half to three hours travelling each day, which did get quite exhausting … The fact that I was doing eight to ten pumps, [the nurses] were very positive, saying, ‘Oh my God, you’ve got a son at home and you’re still managing to do that, that’s amazing!’”* M04.

### Understanding how to sustain and increase milk supply

The majority of mothers had no previous experience of expressing. They valued information from staff about how to maintain or increase their milk supply during long-term expressing (C), including pumping at night, power pumping, eating nutritious foods, taking the milk-stimulating medication domperidone, and keeping a record of their expressing in order to work out the best times.

A few mothers at Units A, C and D commented that they had not been given this information, or nurses made idiosyncratic suggestions, for example *“have sweet desserts with almonds in it and coconut powder”* (M01), without the mother having a way to evaluate the validity of the advice:

*“The nurses, just because they’re also mums, I feel that most of them rely more on their personal experience rather than the correct knowledge…I think maybe they can make an induction package for mums, rather than every time you meet a new nurse she will ask you, ‘Have you tried this?’*” M18.

Several mothers commented that they found it difficult to ask for feeding information unless it was specifically offered. Mothers who had experienced support from specialist infant feeding staff at Units A and B found this helpful: these staff had greater expertise, they took the initiative in checking on progress and offering support, and the mothers did not feel guilty taking staff time away from looking after the babies, as they did with regular nurses.

*“Mums can sometimes be too nervous to ask … [The specialist] would check in on us and see how we were doing and asked what she could do to help. It was very proactive… The nurses were looking after three or four babies at a time, so it was nice to go, ‘Okay, I’m not going to put pressure on the nurses, I’ll go and speak to the specialist who is free and around to talk about this particular subject.’”* M04.

However, mothers at Units A and B could not always access this specialist support when they needed it.

*“There was no set place where the breastfeeding women were, they were just floating, so you don’t know where to find them, it’s just if they happen to come into your room you’d collar them. I don’t even know if there was a breastfeeding specialist there every day.”* M03.

### Neonatal unit facilities and equipment

Mothers had varied opinions of the quality of the neonatal unit facilities available for expressing (O), and the extent to which this influenced their ability to relax and produce milk. This included whether it was practical and comfortable to express next to the baby, and the way that the designated ‘expressing room’ was set up.

*“I found [expressing beside the cot] all rather uncomfortable, it was quite a big set up operation to do that. You had to get a screen and then go and get the pump. I preferred going into the expressing room and doing it there. It was a much more quiet, relaxing space rather than having all the noise and everything else going on around you.”* M19.

The following two mothers reacted positively and negatively to the same expressing room at Unit D, indicating the importance of involving a range of parents in discussions about facilities:

*“The expressing room was quiet … the lights could change colour so there was a certain mood in the room, and there was always soft music playing in the background, so it was okay, they made it as comfortable as they could.”* M23.

*“There was an expressing room on the unit with three pumps and three chairs, and all of the chairs were facing the wall…It has connotations of facing the wall because you’ve been naughty. There was nothing to look at, you were literally staring at a wall while doing the most boring thing imaginable, and if there was somebody else in there that you wanted to talk to, you couldn’t look at them. It was a bit, maybe undertones of shame?”* M22.

All four neonatal units had electric double breast pumps available for mothers to use on the ward (O), and three units loaned similar pumps for mothers to use when expressing at home (O). Mothers who lived at home while their babies were on the unit said that having a good quality breast pump at home was essential for them to continue expressing around the clock.

### Maintaining confidence and positive maternal identity

Some mothers said that by following the advice on frequent pumping had they no problem with maintaining a milk supply that met or exceeded their baby’s increasing needs over the long term, including some mothers who had expressed for many weeks following extremely preterm birth. Some had expressed so much milk that they ran out of space in the neonatal unit’s freezer to store it. As noted for starting expressing, this success reinforced the positive feedback loop of maternal confidence and helped to maintain their motivation to continue (M). Mothers were also sustained by their ongoing intrinsic commitment to do whatever was in their power to help their baby (M).

*“For the first few weeks the only thing I could do for her was express. I couldn’t touch her, I couldn’t comfort her or anything, so I was like, ‘If I can at least give her milk then I’m doing something,’ otherwise I felt quite helpless. That is what kept me going, and then once I saw my supply going up, that was the motivation for me.* I thought *‘We’re getting somewhere, I just need to keep pushing through with it.’”* M04.

### Feeling encouraged in the face of difficulties

For the many mothers who found it difficult to maintain their milk supply, praise and encouragement from health professionals was very important to sustain their motivation in the longer term (M). Sensitive affirmation from staff encouraged mothers to continue pumping even if the volumes produced were small, with a positive focus on what they were able to do rather than what they were not able to achieve:

*“I never really did get much milk … They never doubted me, they never put me down, they always encouraged regardless of whether I got 1ml, 10ml, 100ml, 1000ml, they were always like, ‘That’s great, what you’ve got is great, that’s going to help your baby… you’re doing as much as you can.’ They were the most important words I could have needed at that time. The praise, I suppose, is what I’m getting at.”* M03.

Where a mother was highly motivated and diligent at pumping but unsuccessful at establishing a sufficient breast milk supply, she might become demoralised and give up completely. Fear and stress continued to make mothers vulnerable (M/C). In particular, some who were finding expressing challenging were dispirited when they compared their milk production to others. Positive reinforcement from staff was essential for maintaining mothers’ morale in the face of these comparisons (M).

*“It did get me down at times, I’d maybe have 30ml of milk, and then you’d see women coming in with 200ml of milk and you’d be like, ‘Oh!’ …It’s really soul destroying as an individual who wants so badly to get breast milk and can’t … I didn’t feel like I was doing a very good job as a mum because I wasn’t able to get that milk, but the nurses were always so reassuring and really praised me for how much I just kept going and going and going.”* M02.

It was also important to hear that difficulty with maintaining milk volumes through expressing did not necessarily mean a mother could not achieve her eventual goal of directly breastfeeding (M).

*“When your baby’s in one of those units you’re frightened of everything, and things get blown out of proportion … So telling me, ‘Just because you’re not able to express a lot of milk, doesn’t mean that you won’t be able to breastfeed him.’ Telling me that was very important because it kept me trying… it made me feel like it was something that was still achievable.”* M20.

Other mothers had been relieved to be given ‘permission’ to prioritise self-care when they were exhausted from their demanding schedule of visiting and pumping (C).

*“If it was to a point where you were tired and you couldn’t feed, they will say, ‘Don’t worry, you get your rest… go eat so that you know that you’ve enough that you can feed baby.’”* M23.

One mother who had not had this reassurance had driven herself to the point of breakdown between trying to care for a child at home and her baby on the neonatal unit.

*“I have come [to the unit] today at 7 o’clock until 10 … then I come back again at half one, then I stay here until 4, and my husband goes to work from 5 to 9 …then 10 o’clock I come tonight and then I’ll stay until 12. So, I only hardly get four or five hours sleep…I drove myself for two months, two months I expressed, expressed, expressed, but it went to the point where it was so much for me.”* M01.

### Feeling accountable and honest feedback

A couple of mothers expressed conflicting feelings about receiving consistently positive feedback, which they described as potentially insincere or patronising while also acknowledging that they had benefited from this affirmation (M):

*“They always make you feel really positive and things are going really well, and I sometimes used to think, ‘Are they just saying that and it’s not particularly going well but they’re just wanting to keep my confidence up, or is that genuine?’*” M19.

One mother described how it had been important for her motivation that staff had not just advised her on the frequency of expressing needed, but had kept her feeling accountable by checking on how often she was actually doing it (M):

*“They do establish a level of accountability. The fact that you’re coming back to ask me whether I’ve done what I’ve agreed to do is more than incentive to do it … If they didn’t come round checking I don’t think that I would be doing it as much.”* M08.

Some mothers said that it was important to balance praise with honest feedback about how close they were to matching their baby’s increasing needs, because they could use this information to motivate themselves to keep up with the increasing volume required (M). One described how she was given clear, personalised, non-judgemental feedback, which helped her overcome the impulse to exaggerate how much she was producing and how often she was expressing:

“*[The specialist nurse] goes, ‘How much are you getting?’ I told a slight fib because I didn’t want to look bad, but I was like, ‘Probably about 50ml each time.’ So she calculated it and she goes, ‘You’re really meeting the requirements for now, but you need to be aiming for over 400ml a day within your eight times… I go, ‘Actually, it’s slightly less than what I told you to make me look a bit better.’ So it’s like I really need to get my game on now, I really need to try harder …They are very, very motivating, they’re very supportive and they give you the right information.”* M09.

## Transition to direct breastfeeding

### Understanding how to start breastfeeding

All but one of the mothers wanted to transfer from tube feeding to direct breastfeeding (M). This was a key transition which raised new challenges, including understanding how to establish breastfeeding (C). The first issue was being made aware of their baby showing sucking or feeding cues, with mothers’ experiences ranging from being well-supported to having to work it out for themselves (particularly at Units C and D). The following experiences at Unit C illustrate the inconsistency of support offered for this:

*“[My baby] started wanting to breastfeed about a week ago so they said, ‘Try him.’ … So we started trying him for a couple of minutes a day… and they’d come round just to check that he was latching properly.”* M11.

*“None of the nurses asked me about do I want to breastfeed… I feel like maybe they think all mums are there to basically feed their babies in a bottle and get them home.”* M10.

Most mothers needed information and support with the practical side of introducing their very preterm baby to breastfeeding, even if they had experience of breastfeeding other children. In particular, mothers valued skilled support to assess whether the baby was feeding effectively. Some (at all units) had received excellent support.

*“When I first started, the nurse watched and she was like, ‘I can see she’s getting something because I can see she’s swallowing, and when she opens her mouth I can see milk in there.’ So that was really nice to hear and see, because I always used to say, ‘I can’t feel it coming out.’*” M03.

Others (at all units) had received no support at all to establish breastfeeding, and in some cases this had led to the mother giving up. Mothers again highlighted the importance of feeding support from staff who were not also juggling responsibilities to care for the babies.

*“I’ve not really had any support, to be honest. No one has spoken to me about it or anything… It’s getting to the point where I’m trying him on the breast every once in a while, and I’ve absolutely no idea if I’m doing it correctly.”* M16.

“*I maybe should have asked for help, but when I can see that they’re working under pressure and they’re busy, I didn’t want to be that nuisance mum…When I know a nurse has got to care for three babies and then I say, ‘Can you help me with breastfeeding?’ and she’s with me for 30 min, I felt like I couldn’t do that.”* M02.

Particularly at Units C and D, where there was very limited infant feeding specialist support, mothers reported that if they did take the initiative to ask for help with breastfeeding, they received contradictory advice from staff nurses.

*“Whichever nurse was on shift at the time would help me if I asked her for help. The trouble was that people would give slightly different advice … I basically changed what I was doing depending on what I was told every time because I felt really insecure that I’m not doing the right thing … There was one lady who was a sister but also a specialist in breastfeeding who came and spoke to me … she cleared a lot of things up for me, but then she was never available on the day, so I never saw her again. If I had her or someone else constantly there I would have gone to the same person every time and I think it would have been a smoother experience.”* M21.

Some mothers suggested that systematically providing every mother with basic information about breastfeeding a very preterm baby would be a useful starting point, as well as encouraging all staff to see that supporting a mother to breastfeed (and not just to provide breast milk) was part of caring for the baby.

*“Just consistency amongst the nurses … Their job is to look after the baby, but you could say that breastfeeding is an extension of that … So maybe some sort of information, whether it be written or someone talking to you about this stage. Everything you need to know, even a bloody leaflet or something.”* M17.

### Coping with exhaustion, fear and the baby’s own needs

Mothers continued to express milk for top up tube feeds during the period where they and their babies were learning to breastfeed. They commented on the enormous strain of adding breastfeeds to the already demanding expressing schedule (O).

*“When you’re trying to breastfeed and you’re trying to fit in your pumping at the same time you just feel like there’s no break from it. Before you were only doing the pumping side of it, so that was your commitment every three hours, then you’re trying to fit in a breastfeed in that window between the three hours, it just felt as if it was all about the feeding.”* M19.

The fact that their babies had reached this milestone did not necessarily allay mothers’ anxiety (M), and staff sensitivity to the traumatic nature of the experience remained very important to supporting mothers effectively.

*“Definitely the pressure is on, it feels like a big step but I’m trying not to get overwhelmed by it. Because I’m overwhelmed by so much more. This is going to sound really dark, but is he going to live? …. Probably what often happens in medicine is that something that seems really alarming or difficult to a mum who has only just started doing it, seems really everyday to the nurse, so some of them are a bit matter of fact about it. The ones that you create a bond with, they get it.”* M17.

There were also factors affecting physical capability of the mother-baby dyad that were outside the control of the mother and the staff: for example the baby was not developmentally able to manage sucking, swallowing and breathing, the baby needed breathing support, or the baby lacked the stamina needed to breastfeed for more than a few minutes at a time (C). A couple of mothers gave up trying to breastfeed when it became apparent that their baby would indefinitely need top up feeds through a tube or by bottle, so this was not a transitional stage from expressing to full breastfeeding (M).

“*I only did exclusive expressing, because her lung was not very well. So, we tried breastfeeding around 38 weeks when she was off CPAP [breathing support], but she seemed very tired* … *I need to express before feeding her … and after breastfeeding I need to express again. So, I gave up in three days. It was too difficult.”* M18.

### Spending time on the neonatal unit

Even if a mother was highly motivated to establish direct breastfeeding, the amount of time that she could spend on the neonatal unit was crucial for her to succeed (O), and this was complicated at Units A, C and D by pressure from staff to offer the baby a bottle if the mother was not present on the unit to offer her breast. One mother described being warned by an infant feeding specialist at a previous neonatal unit that she would have to be very robust about her decision not to use bottles at all.

*“She said, ‘What you’ll find is that they’re probably going to push bottles on you.’…I was really pleased that I’d been pre-warned because otherwise I definitely would have given up sooner. I found that even though I had ‘no bottles’ written on my form that I was repeatedly asked, ‘Shall we just give her a bottle?’… I got the feeling that it was more convenient for them … and it would be quicker to get me out the hospital as well.”* M07.

There also could be a tension between the hospital’s schedule for feeding and the reality of introducing breastfeeding during limited visits to the neonatal unit:

*“What was really hard was [my baby] was on a three-hour feeding schedule by tube, and that meant that when I went to breastfeed, he wasn’t always hungry…But as soon as we switched to it being more demand-led, the breastfeeding became a hell of a lot easier.”* M19.

These challenges could be overcome where the unit had a room where the mother could stay with her baby for a few days (O).

*“The nurses were saying that the babies are looking to suck in the middle of the night, so then I was suggested to come in and stay, and they did fabulously throughout the whole night.”* M13.

This option was not offered to all mothers. One mother was bewildered by discharge criteria at Unit C that required 48 h of full oral feeding, which appeared impossible for a fully breastfeeding mother to fulfill without being able to stay at the neonatal unit, and she had not been offered anywhere to stay. She interpreted the criteria as meaning it was compulsory to introduce bottles before discharge:

*“Part of the discharge criteria, they say the baby has to be bottle fed for 48 h, so I was like, ‘What if I want to breastfeed?’ Right, that’s my goal then, isn’t it? I have to bottle feed for 48 h and no other alternative … Well, you can stay in 24 h [to breastfeed] if you wanted to sit on a chair.”* M10.

### Confidence about weight gain and managing top-up feeds

Mothers’ motivation to continue with introducing breastfeeding could also be affected by their confidence about whether breastfeeding was enough for their baby’s needs (M). A couple of mothers had accomplished the transition easily and confidently.

*“He had no problems at all … I was like, ‘Wow, I’ve managed to produce enough milk to satisfy him and he’s fallen asleep and he’s got milk dripping all over his face, and that’s from me.’ I can’t tell you how that felt… When he was weighed the first time after the first week he was 100% fully breastfed and he stayed the same weight, we were cheering*.*”* M20.

Most mothers, however, had experienced challenges related to the natural tendency for a baby’s weight gain to be adversely affected for a short period when the baby was using more energy to feed and was drinking ‘pure’ breast milk rather than being tube fed breast milk that was fortified with human milk factor or mixed with high-calorie premature formula milk. As with other stages of their breastfeeding journey, many mothers said that reassurance about what was normal was essential for their confidence to continue breastfeeding, and some mothers said that their confidence was also helped by peer support and the opportunity to discuss and normalise the challenges with other mothers.

*“It’s a bit discouraging thinking that you’re feeding your child but he’s not getting the nutrients or the calories that he needs from you… [Staff said] it is normal, baby will gain back weight, you have to just keep feeding … Hearing other parents’ ups and downs of trying to breastfeed and trying to make sure that they are feeding enough and whether the babies need top ups, it made you feel like you weren’t the only one.”* M23.

One mother’s experience demonstrated how her confidence in her body’s ability to breastfeed could be dramatically undermined at this point by a health professional who withheld this reassurance.

*“And then an awful experience near the end of the journey where we took her off the tube and I was feeding her and she was losing weight for the first couple of days …[The doctor] basically made it sound like I was starving my child* … *I felt like an utter failure.”* M07.

However, even reassurance was not always enough for a mother whose long neonatal unit experience had led her to focus on weight gain as the most important indicator of her baby’s wellbeing, and who believed that faster weight gain was key to being discharged sooner.

*“For premature babies their weight is the most important thing for parents, because we don’t understand other figures or more complicated medical terms, so the weight is one of the easiest things we can understand…. If she is thriving, she can go home earlier, if she gains weight enough maybe she will be off oxygen earlier… And if she stopped gaining weight, [it felt like] I failed her.”* M18.

This challenge was closely linked to mothers’ anxiety about quantifying how much milk their baby was taking from the breast compared with precise measurement of volume given by tube or bottle, and how to manage the additional top-ups (via tube or bottle) that might be needed.

*“The whole situation is very medicalised, so it’s every three hours on the dot and they work out the volume based on mls per kilogram, so you might be giving 23 mls and, it went up from 21 and you’re pleased that it’s gone up 2 mls, and it’s very precise and very accurate. And then when you introduce breastfeeding all of that accuracy goes out of the window. I felt like I had to hold my nerve and have the confidence to not top him up too much … The more top-ups we gave him the less he would breastfeed because he wouldn’t be hungry.”* M22.

Rigid application of a structured policy for calculating the amount of top-up feeds was not necessarily supportive for mothers and babies who were trying to learn how to breastfeed responsively.

*“They have a chart that they follow, and they would say if they’ve [breastfed] less than 10 min then you must give them a top-up a bit later to make sure that they’re full, and I feel like whenever I did that they would spit out a lot of milk…They’re wet and you have to change them, so that’s making them cold. …They’re crying so much it’s tiring them out, because we’re disturbing them every three hours when they’re sleeping, taking them out of the cot and then trying to feed them when they’re probably not hungry, because if they are hungry they would wake up…So maybe [the unit should] give you the time to breastfeed them the way you would want to feed them.”* M05.

## Going home breast milk feeding

### Feeling confident to go home breast milk feeding

Of the 13 mothers whose babies had been discharged at the time of the interview, five had exclusively breastfed at discharge, one had exclusively expressed, four had combined breastfeeding and expressing, two had combined expressing and formula milk, and one had exclusively formula fed (having actively chosen to stop breast milk feeding before discharge).

These diverse feeding strategies reflected mothers’ and babies’ needs and capabilities and required different forms of preparation. Mothers particularly appreciated it when staff were able to spend the time ensuring that they were realistically prepared to the practicalities of their feeding situation when they were home, so they were confident to manage this (M). Their feeding confidence had been consolidated by being able to spend at least 24 h rooming in with their babies at the neonatal unit as a routine part of discharge (O), irrespective of feeding method.

*“I really appreciated having those couple of nights rooming-in because I found out, by breastfeeding through the day, by night time she was exhausted so we just did full tube feeds, didn’t even try and breastfeed because she was just too tired….If I would have come home with her straightaway and she would suddenly in the night not be feeding and stuff, I’d be panicking.”* M15.

Several mothers said that knowing they would have ongoing support for feeding through the neonatal unit’s community outreach team (at Units B and D) had also helped their confidence in taking their baby home breastfeeding or breast milk feeding.

*“They said that we could go home on Saturday and then we weighed him on the Saturday and he’d lost weight …I said to the doctor, ‘I don’t see what it achieves by us staying here because we’ll just do the same things if we’re at home,’ and they have a brilliant homecare team who I already knew from on the ward …and he said, ‘Okay, well just give him a few top ups.’”* M22.

### Sustaining breast milk feeding after discharge

Some mothers who came home fully or partly expressing said that they had not been able to sustain the pumping once they were home and looking after their baby full time (C). Mothers who had built up a store of frozen expressed breast milk might use it up at this point, or they might introduce formula milk to supplement or replace the expressed breast milk.

“*My milk supply dropped even more when I came home as well because I wasn’t being able to express those eight times in the day, I was probably only expressing twice a day by that point … I think my body just thought I wasn’t needing that demand anymore and it went from poor to really poor.”* M02.

By contrast, some mothers who were fully breastfeeding at the point of discharge found this sustainable, and even more successful once their babies had left the neonatal unit and could be fed responsively (C).

*“Ever since I have brought them home, it’s not tiring at all for me to respond to them whenever they’re hungry, and I just quickly put them on my breast and then maybe they will take 10 or 15 min and they will just go to sleep immediately, so it’s less work for me.”* M05.

Some of the mothers who came home feeding breast milk said that their baby’s weight had dropped after leaving the neonatal unit when the baby was no longer being given human milk fortifier added to the milk, or within a few weeks they could not keep up with the baby’s increasing needs. Ongoing support from the neonatal unit outreach team was very important to manage this situation with personalised advice, usually about supplementing the breast milk with infant formula, or ‘permission’ to stop breast milk feeding.

*“Obviously I did want to carry on feeding her, but [also] to try and soak up all those moments with her rather than prioritise trying to express the little amount that I was getting… I just needed someone else to say, ‘It’s okay, you can stop.’ I didn’t want to feel like it was just my decision.”* M03.

## Discussion

Using the COM-B framework to analyse mothers’ experiences has enabled exploration of the interrelationships between mothers’ capacity, opportunity, motivation and behaviour across five time points on the breast milk feeding journey in the neonatal unit. Although the issues mothers raised were generally in line with previous qualitative studies about breast milk feeding in neonatal units for babies of varied gestations [20–23], this study identifies how the interacting ways in which these issues affected motivation, capability and opportunity could support or obstruct the behaviours of breast milk feeding and breastfeeding. Information from staff about the benefits of breast milk for very preterm babies had motivated all the mothers to begin expressing, but fear of forming an attachment to a baby who might die could undercut mothers’ motivation. The trauma of very preterm birth was an obstacle to mothers’ capability to engage with information about the benefits and the practicalities, both before birth and in the immediate aftermath, which is the critical time window for stimulating lactation.

In addition to the physical challenge of their own recovery after the medical emergency of very preterm labour or caesarean section, there were further obstacles to mothers’ capability to begin expressing if they were not shown how to express or were given contradictory information by postnatal ward staff and neonatal unit staff, with confusion over who was responsible for supporting the mothers to express, as also reported by Wilson [32]. Success at expressing colostrum motivated mothers to feel this was something their bodies could do well, even if they felt guilty that their bodies had ‘failed’ to keep their babies safe to a full term birth. Swanson et al. [24] describe failure to establish a milk supply as reinforcing mothers’ negative self-schemas activated by preterm birth. Giving mothers the motivation to express breast milk but not supporting their opportunity or capability could lead to demoralisation and self-reproach. However, if early attempts at expressing were unproductive, staff could sustain mothers’ motivation through encouragement and reassurance that stimulating the breasts at this very early stage was a positive step in itself, irrespective of milk volume.

Mothers of very preterm babies face the specific challenge of sustaining milk supply through expressing for weeks or months, and previous research has drawn attention to the psychological difficulty of replacing the intimate breastfeeding relationship with a mechanised process that both distances them from and links them to the baby [33]. Self-confidence as a mother, and a sense of expressing as a core part of maternal identity while on the neonatal unit, were motivations that reciprocally affected and were affected by ongoing expressing in a positive feedback loop, as described by Swanson et al. [24] for preterm babies more generally. However, determined motivation to continue long term expressing was not enough if mothers lacked capability and opportunity. Where mothers encountered difficulties in maintaining and increasing their milk supply in line with their babies’ increasing needs, accurate and consistent information from staff was again key, alongside sensitive and non-judgemental feedback and affirmation as previously reported [20–23], even if some questioned the authenticity and diminishing returns of constant praise. Some also valued ‘permission’ to take a break from expressing when they were exhausted. In contrast to teenagers with preterm babies in the USA [34], the adult mothers in this study responded well to accountability where well-informed specialist staff asked directly but non-judgementally about how much and/or how often they were expressing, in order to give personalised support to meet their aspirations for future feeding.

Unlike the comprehensive peer support strategy reported by Meier et al. [35] and the peer counsellors reported by Rossman et al. [36], in the wake of the Covid-19 pandemic there was no organised peer support at these units. Although some mothers benefited from informal peer support from other parents in normalising challenges, others might become demoralised by peer comparison about the volume of milk they were expressing, reinforcing the suggestion made by Li et al. [22] that opaque milk containers could be used. Family Integrated Care, in which parents are supported to be their babies’ primary caregivers on the neonatal unit, is associated with increased high-frequency breastfeeding at discharge for very preterm babies [37]. None of the units had facilities for parents to stay with their babies in single family rooms (except immediately before discharge), which can facilitate Family Integrated Care [38] and support the transition to direct breastfeeding [10, 17, 18]. Some mothers were able to make use of nearby accommodation provided by the hospital and spent extended periods of time holding their babies skin-to-skin and expressing, while others lived at home and had to divide their time between expressing, travelling, visiting their baby, and caring for other children. These experiences generated different levels of physical opportunity for expressing and breastfeeding, indicating that future design of neonatal units should aim to minimise separation of mothers and babies where feasible.

Not all babies were able to directly breastfeed and not all mothers wanted to; others wanted to breastfeed but lacked confidence about how much milk a baby was taking and worried about weight gain. These concerns could be exacerbated or ameliorated by staff, and mothers also relied on staff for information about recognising feeding cues and how to introduce a very preterm baby to the breast, which was not always forthcoming. Mothers’ physical opportunity and ability to spend time on the neonatal unit was critical for their ability to establish breastfeeding, and in mothers’ absence, there could be pressure from staff to introduce bottles for a baby who was ready for oral feeding. There were no reports of staff unilaterally acting without mothers’ consent as reported by Mӧrelius et al. [39], but they could create an atmosphere of implied criticism that was difficult to resist.

Whether their babies were discharged breastfeeding, tube feeding, bottle feeding, or a combination, mothers valued ongoing support from the neonatal unit’s community outreach team where this was available. Consistent with studies about the length of time very preterm babies are fed breast milk after discharge [10, 16], the mothers who were exclusively breastfeeding at discharge had tended to continue, while the mothers who were expressing had tended to give this up after a short time, their motivation defeated by the physical difficulty of maintaining a schedule of expressing and feeding as well as caring for their baby full time, in addition to (for some) looking after other children.

This study was carried out at four neonatal units with different levels of specialist staffing for feeding support, and some mothers described being helped by specialist staff through their proactive approaches, and their knowledgeable, empathetic and skilled support. Mothers felt that it was legitimate to use specialist staff time on feeding issues when this was not a busy nurse looking after several babies. Although these positive reports were concentrated in the units that had the higher levels of specialist staffing, some mothers said that even at these units they were not always able to access the specialist when they needed support. Mothers at all four units also reported negative experiences of feeling judged and undermined, and being given incorrect information about aspects of the feeding journey, and in every case, this was from the wider neonatal or maternity teams. This reinforces the importance of training the whole team to be effective promoters and supporters of breast milk feeding [35], as well as investing in specialist support and leadership [3, 40–42]. It also highlights the need for all staff to be mindful of parents’ vulnerability and dependence in the unfamiliar and anxious situation of a neonatal unit, and to bring a trauma-informed approach to all interactions with parents, so that staff understand the impact of past and present trauma and how it can affect a person’s response to situations, are aware of the signs of trauma responses, and actively work to prevent retraumatisation [43]. In the context of feeding, this means acknowledging the traumatic situation of very preterm birth, giving information and discussing choices with clarity and empathy and at times and in ways that mothers can engage with, and understanding that mothers of very preterm babies may be very sensitive to implied criticism as they try to navigate their feeding journey [24, 39]. A wide range of interventions have been tried to improve the psychosocial wellbeing of parents with babies in neonatal care, but it is not clear which are most effective or applicable for parents of very preterm babies [44].

Previous research has emphasised the need to provide mothers with accurate and realistic information about breast milk feeding, written as well as verbal, repeated as often as needed [22, 39], and parents in this study echoed the need to be have a standardised pack of information about feeding to refer to, as well as proactive personalised advice. Staff also need to be able to balance championing breast milk feeding with sensitive support for mothers who cannot maintain a milk supply despite their commitment, and to be able to judge when a mother needs encouragement to continue and when she is really asking for ‘permission’ to stop. A key point is to focus affirmation on mothers’ efforts and commitment, rather than on the volume of milk she manages to produce.

### Strengths and limitations

It was a strength of this research that the participants were demographically diverse mothers with a range of breast milk feeding experiences, from four neonatal units with different models of support and different rates of very preterm babies being fed breast milk at discharge. Using a COM-B framework enables reflection on the different aspects of support needed at different time points. It was a limitation that the small number of mothers interviewed at each unit does not enable us to draw firm inferences about impact of the different models of support on mothers more generally. Only two mothers with twins participated, and more research is needed on the specific feeding challenges faced by mothers with very preterm multiple births.

## Conclusion

Interventions to increase breast milk feeding for very preterm babies on neonatal units should address mothers’ motivation, capability and opportunity, aiming for systematic elimination of obstacles at each stage of the feeding journey. Mothers value personalised and skilled specialist support, but also need other staff to be able to give consistent, accurate information and affirmation focused on their efforts rather than their success, and to take a trauma-informed approach in their interactions. Investing in rooming-in facilities that minimise the separation of mothers and babies where feasible, is likely to overcome a key obstacle to breast milk feeding and the transition to direct breastfeeding.

## Supplementary Information

Supplementary Material 1.

## Data Availability

The datasets generated during the current study are not publicly available due to the consent process but are available from the corresponding author on reasonable request.
