# Supplementary material for: What supports mothers of very preterm babies to start and continue breast milk feeding neonatal units? A qualitative COM-B analysis of mothers’ experiences
Source: BMC Pregnancy Childbirth. 2024 Nov 6;24:725. doi: 10.1186/s12884-024-06910-4 (PMC11542208; doi:10.1186/s12884-024-06910-4)
Supplement: Supplementary file 1 — Supplementary Material 1. [file 12884_2024_6910_MOESM1_ESM.docx]

**Breastfeeding very preterm babies in neonatal units: topic guide – mothers**

1. Demographic questions: mother’s age, occupation, ethnicity, other children. Baby’s DOB, gestational age at birth, any diagnosis, when discharged from NICU.How they are doing now.
2. Feeding expectations or plans before birth (incl how other children were fed, family feeding culture, antenatal visit to NICU, information about feeding)
3. Any rules or restrictions re: covid at the NICU? Impact?
4. Tell me about how feeding has been since the baby was born
   1. getting started
      1. where/when?
      2. Who supported?
      3. Offered opportunity to express colostrum just before birth?
   2. expressing /tube feeding
      1. how long did it last
      2. where/ how often/ how did you do it (e.g. cot side or in parents’ room)
      3. who supported?
      4. role in tube feeding?
      5. what did you feel about expressing/tube feeding?
   3. Breastfeeding
      1. how long did it last?
      2. Transition tube > BF
      3. Who supported it?
      4. What did you feel about breastfeeding?
      5. What was it like stopping?
   4. Mixed feeding/supplementation?
      1. How did this work?
      2. Was there any information/support to enable you to move to fully breastfeeding?
      3. How did you feel about it?
   5. going home
      1. Feeding method at that point? (any pressure to use bottles in order to go home?)
      2. How did this work?
      3. Who supported?
      4. Did you have enough information about feeding at home
      5. How did you feel about it?
5. What helped you to keep going?
6. What made it harder?
7. What’s the one thing you would change about how breastfeeding and breastmilk feeding are supported on the NICU, if money were no object
